# The clinical characteristics of pleural effusion in scrub typhus

**DOI:** 10.1186/s12879-016-1613-0

**Published:** 2016-06-11

**Authors:** Hyung Ho Kim, Jong-Hoon Chung, Dong-Min Kim, Na Ra Yun, Jun Lee, Yong Eun Kwon, Sung Ho Yoon, Seung Il Lee, Mi Ah Han

**Affiliations:** Department of Internal Medicine, Chosun University College of Medicine, 588 Seosuk-dong, Dong-gu Gwangju, 501-717 South Korea; Department of Preventive Medicine, Chosun University College of Medicine, Gwangju, Republic of Korea

**Keywords:** Scrub typhus, Pleural effusion, Korea

## Abstract

**Background:**

The aim of this study is to identify the factors associated with the occurrence of pleural effusion and to investigate the characteristics of pleural effusion in scrub typhus.

**Methods:**

We conducted a retrospective analysis of the medical records of scrub typhus patients between January 2004 and December 2011 at Chosun University Hospital in South Korea. A total of 445 scrub typhus patients were divided into the following two groups: without (*n* = 352) or with pleural effusion (*n* = 93). The data of 18 scrub typhus patients who underwent thoracentesis were summarized.

**Results:**

Multivariate analysis demonstrated that the following factors were associated with the occurrence of pleural effusion in scrub typhus: older age (odds ratio [OR] = 1.029, *P* = 0.037, confidence interval [CI] = 1.002–1.056); male gender (OR = 1.924, *P* = 0.020, CI = 1.109–3.340); presence of heart failure (OR = 2.628, *P* = 0.039, CI = 1.052–6.565); and lower albumin (OR = 0.107, *P* ≤ 0.001, CI = 0.058–0.196). Most pleural effusion presentations were bilateral (88 %) and small (91 %). The effusion had transudate characteristics in 7 patients and exudate characteristics in 11 patients based on Light’s criteria.

**Conclusions:**

This study provided the first data regarding the following four independent risk factors associated with the occurrence of pleural effusion: older age; male gender; the presence of heart failure; and lower albumin. The pleural effusion presentations in scrub typhus patients were bilateral and small in most cases, with transudate and/or exudate characteristics.

## Key points

This study provided the first data regarding the following independent risk factors associated with the occurrence of pleural effusion: older age; male gender; the presence of heart failure; and lower albumin.

## Background

Scrub typhus is a mite-borne infectious disease of humans caused by *Orientia tsutsugamushi* [1], and it is characterized by widespread vasculitis or perivasculitis in multiple organs including the lungs, cardiovascular system, central nervous system, kidneys, gastrointestinal tract, liver, spleen and lymph nodes [2–4]. Information regarding pulmonary involvement in scrub typhus infection is comparatively well-documented, including data regarding interstitial pneumonia, pulmonary alveolar edema, hemorrhage, consolidation, atelectasis, cardiomegaly and pleural effusion [5–9]. Few studies include results on the incidence of pleural effusion, and there have been no studies on the characteristics of pleural effusion. This study was conducted to identify the clinical and laboratory factors associated with the occurrence of pleural effusion and to investigate the characteristics of pleural effusion scrub typhus in adults.

## Methods

We conducted a retrospective analysis of the medical records of confirmed adult scrub typhus patients (age ≥ 18 years) between January 2004 and December 2011 at Chosun University Hospital in the southwestern region of South Korea.

The diagnosis of scrub typhus was confirmed when an indirect immunofluorescent antibody assay (IFA) IgM titer against *O. tsutsugamushi* increased to ≥ 1:80, an IFA titer against *O. tsutsugamushi* increased four times or more, or when a polymerase chain reaction result was positive [10]. The confirmed diagnosis of scrub typhus was made in 445 patients. Pleural effusion was observed on chest radiographs in 93 of these patients, and pleural fluid examination was performed in 18 patients.

**Fig. 1:**
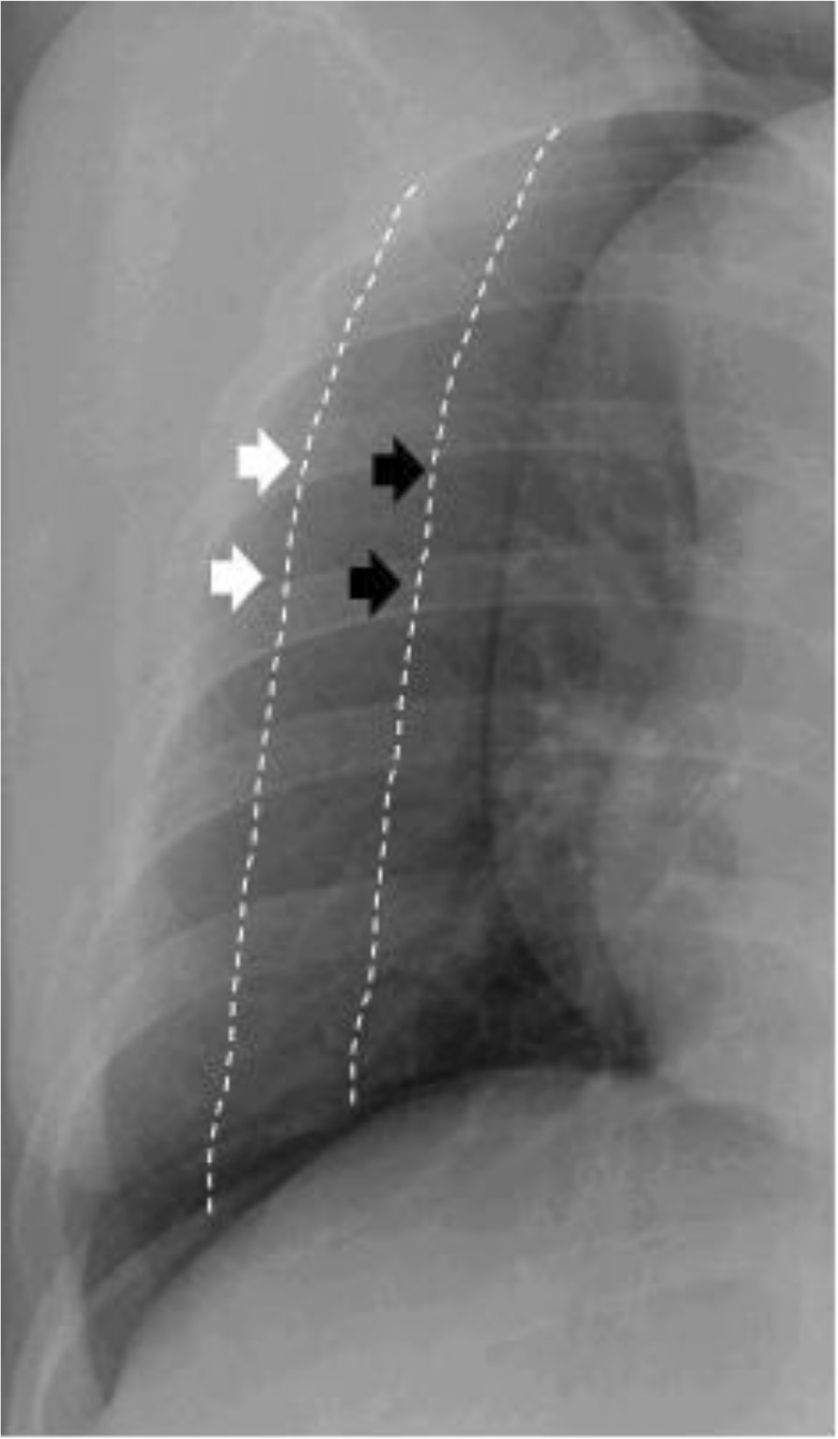
Semiquantification of the size of the pleural effusion (white arrow = small effusion, black arrow = modeate effusion)

The medical records and radiographic studies of the included patients were reviewed for the analyses. Data were collected regarding the demographic characteristics [including age, gender and medical history], the initial clinical symptoms/signs [including fever, cough, sputum, dyspnea and rash], laboratory findings [including the peripheral WBC count, hemoglobin level, platelet count, aspartate aminotransferase (AST) and alanine aminotransferase (ALT) levels, alkaline phosphatase (ALP), serum total bilirubin, serum creatinine (Scr), albumin, erythrocyte sedimentation rate (ESR), C-reactive protein (CRP) and lactate dehydrogenase (LDH) levels], and pleural fluid laboratory findings. The pleural fluid data [including the pH, WBC count, monocyte ratio, total protein, LDH, albumin, glucose, adenosine deaminase (ADA) and carcinoembryonic antigen (CEA) levels], size and location were collected for the patients who underwent thoracentesis. The size of the pleural effusion was semiquantified on the basis of the decubitus radiographs by visually estimating the area of the hemithorax that was occupied by pleural fluid as follows: no effusion = no pleural fluid present; small effusion = pleural fluid occupied less than one third of the hemithorax; moderate effusions = pleural fluid occupied between one third and two thirds of the hemithorax; large effusion = pleural fluid occupied more than two thirds of the hemithorax (Fig. 1). The effusion was considered bilateral if present on both sides. Patients with bilateral effusion had a size classification based on the larger of the two sides on a chest radiograph. The pleural fluid was characterized as a transudate or an exudate based on the criteria described by Light *et al* [11]. The following criteria were evaluated: a pleural fluid (PF)-to-serum (S) protein ratio > 0.5, a PF-to-S LDH ratio > 0.6, and PF LDH > two-thirds of the upper normal limit for the S LDH (the cut-off value of PF LDH was 140.6 in 2008–2009 [reference range, 106–211 IU/L, the L-lactate-to-pyruvate method using SICDIA L LDH (J) and Hitachi 7600, Hitachi, Japan] and 306.6 IU/L in other years [reference range, 180–460 IU/L, the pyruvate-to-L-lactate method using SICDIA L LDH and TBA-C8000, Toshiba, Japan]).

The data were collected and analyzed by SPSS for Windows version 18.0 (SPSS, Inc., Chicago, IL). The continuous variables were expressed as the mean ± standard deviation, and the comparisons of the means between the two study groups were determined by Student’s *t*-test for the continuous variables and a Chi-square test/Fisher’s exact test for the dichotomous variables. To determine the factors associated with the occurrence of pleural effusion in scrub typhus, a multivariate logistic regression analysis was performed using a forward stepwise model with the independent variables having a *P*-value of <0.05 that were obtained by a univariate analysis. The odd ratios with 95 % confidence intervals are presented. A *P*-value of <0.05 was considered to be statistically significant.

## Results

### The demographics, clinical characteristics and laboratory findings of scrub typhus patients

A total of 445 confirmed adult scrub typhus patients were divided into the following two groups: without pleural effusion (*n* = 352) and with effusion (*n* = 93). The demographic characteristics, clinical presentations and laboratory findings at admission are summarized in Table 1. The mean age of the group with effusion was 70.5 ± 10.7 years, and 45 patients (49.5 %) in this group were male; these numbers were greater than those for the group without effusion. The group with effusion had a significantly higher percentage of underlying comorbidity with hypertension, heart failure and liver cirrhosis and symptoms of cough and dyspnea. However, the presence of rash was lower than that of the group without effusion. Additionally, there were significant differences in WBC count, hemoglobin, ALP, total bilirubin, total protein, albumin, serum creatinine, ESR and CRP levels between the two groups. These variables were significantly correlated with the occurrence of pleural effusion in the univariate logistic regression model. Input variables for the multivariate model were selected from significant variables obtained from the univariate analysis. Cough and dyspnea were not included because these symptoms could be presented after pleural effusion occurred, and protein, ALP, ESR and CRP were also not included to eliminate the influence of multicollinearity. Because both backward and forward model selection methods produced similar results, data from the forward stepwise model are presented. From this mutivariate model for the occurrence of pleural effusion in scrub typhus, only four factors remained as independent risk factors: older age (OR = 1.029, *P* = 0.037, CI = 1.002–1.056); male gender (OR = 1.924, *P* = 0.020, CI = 1.109–3.340); presence of heart failure (OR = 2.628, *P* = 0.039, CI = 1.052–6.565); and lower albumin (OR = 0.107, *P* ≤ 0.001, CI = 0.058–0.196) (Table 2).

**Table 1:** Demographic, clinical characteristics and laboratory findings of confirmed adult scrub typhus patients

Characteristics	Without PE (*n* = 352)	With PE (*n* = 93)	*p* value
Demographic characteristics			
Age, mean years ± SD *	61.4 ± 14.0	70.5 ± 10.7	<.001
Male gender (%) *	109 (31)	46 (49.5)	.001
Underlying comorbid condition *	134 (38.1)	49 (52.7)	.011
Hypertension*	80 (22.7)	33 (35.3)	.012
Diabetes mellitus	35 (9.9)	14 (15.1)	.161
Chronic kidney disease	0 (0.0)	1 (1.1)	.209
Congestive heart failure *	20 (5.7)	11 (11.8)	.038
Liver cirrhosis *	5 (1.4)	7 (7.5)	.004
Chronic obstructive pulmonary disease	10 (2.8)	1 (1.1)	.472
Malignancy	7 (2.0)	3 (3.2)	.442
Time from disease onset to hospital visit, mean days ± SD	7.50 ± 6.29	7.99 ± 6.91	.516
Clinical symptoms (Initial presentation)			
Fever	340 (96.6)	88 (94.6)	.368
Cough *	85 (24.1)	32 (34.4)	.046
Sputum	57 (16.2)	19 (20.4)	.334
Dyspnea *	45 (12.8)	28 (30.1)	<.001
Rash *	303 (86.1)	71 (76.3)	.023
Laboratory findings			
WBC count (no. of cells × 1,000/mm^3^) *	7.5 ± 3.6	10.3 ± 4.3	<.001
Hemoglobin (g/dL) *	12.7 ± 1.7	12.0 ± 1.7	.001
Platelet count (no. of cells × 1,000/mm3)	152.2 ± 67.6	150.0 ± 87.2	.823
AST (IU/L)	126.3 ± 299.4	155.9 ± 308.1	.401
ALT (IU/L)	93.1 ± 107.6	98.6 ± 154.2	.690
ALP (U/L) *	115.1 ± 90.5 (*n* = 348)	157.1 ± 141.7 (*n* = 92)	.008
Bilirubin (mg/dL) *	0.84 ± 0.71	1.20 ± 1.19	.006
Protein (g/dL) *	6.40 ± 0.70	5.77 ± 0.77	<.001
Albumin (g/dL) *	3.75 ± 0.48	3.11 ± 0.51	<.001
> 3.5, no. of patients (%)	248 (70.5)	23 (24.7)	
3.0–3.5, no. of patients (%)	81 (23.0)	25 (26.9)	
≤ 3.0, no. of patients (%)	23 (6.5)	45 (48.4)	
Serum creatinine (mg/dL) *	1.08 ± 0.49	1.45 ± 0.95	<.001
ESR (mm/h) *	18.6 ± 15.3 (*n* = 350)	24.6 ± 22.2 (*n* = 92)	.016
CRP (mg/dL) *	8.0 ± 7.2 (*n* = 351)	10.7 ± 5.7 (*n* = 93)	.001
LDH (U/L)	762.8 ± 346.8 (*n* = 346)	736.4 ± 431.1 (*n* = 92)	.538

*Abbreviations*: *AST* aspartate aminotransferase, *ALT* alanine aminotransferase, *ALP* alkaline phosphatase, *LDH* lactate dehydrogenase, *ESR* erythrocyte sedimentation rate, *CRP* C-reactive protein

* There was a significant difference in the baseline characteristics between the two groups (P < .05)

**Table 2:** Univariate and multivariate logistic regression analysis of the occurrence of pleural effusion in scrub typhus

	Univariate	Multivariate	
	OR (95 % CI)	OR (95 % CI)	*P* value
Age, mean years ± SD	1.066 (1.041–1.091)	1.029 (1.002–1.056)	0.037
Male gender	2.182 (1.370–3.474)	1.924 (1.109–3.340)	0.020
Presence of heart failure	2.227 (1.027–4.831)	2.628 (1.052–6.565)	0.039
Albumin (g/dL)	0.082 (0.047–0.145)	0.107 (0.058–0.196)	<0.001

*Abbreviations*: *OR* odd ratio, *CI* confidence interval

### Chest radiography of scrub typhus patients with pleural effusion

During the study period, radiographic evidence of pleural effusion was noted in 93 patients (20.9 %) at admission. Among them, 69 patients (74.2 %) had radiograph re-examinations during the first week of hospitalization by attending physicians, and progressive changes (31/69) were frequently observed. A total of 37 patients had a more than 10-mm thickness of pleural fluid on the decubitus radiograph (39.8 %), and pleural fluid examination was performed in 18 patients. Most of the effusion presentations were bilateral (88 %) and small (91 %), and the exact locations and sizes are presented in Table 3.

**Table 3:** Chest radiography of the scrub typhus patients with pleural effusion (93 patients)

Variables	No. (%)
Detected effusions	
Day 1	93 (100)
Day 2–7	
No study	24 (26)
Progressive	31 (33)
Not progressive (no change or Improved)	38 (41)
> 10-mm thickness on the decubitus radiograph	37 (40)
Performed thoracentesis	18 (19)
Location	
Unilateral right sided	8 (9)
Unilateral left sided	3 (3)
Bilateral	82 (88)
Size	
Small^a^	85 (91)
Moderate^b^	7 (8)
Large^c^	1 (1)

^a^ Pleural fluid occupied less than one-third of the hemithorax

^b^ Pleural fluid occupied between one-third and two-thirds of the hemithorax

^c^ Pleural fluid occupied more than two-thirds of the hemithorax

### Characteristics and laboratory findings of pleural effusion in scrub typhus

The characteristics and laboratory findings of pleural effusion in scrub typhus patients who underwent thoracentesis are presented in Table 4. The mean days from the initial symptom presentation to the first thoracentesis was 13.17 ± 13.11 days, and the mean days from admission to the first thoracentesis was 2.50 ± 2.57 days. The examined pleural fluid was located bilaterally in all (100 %) the patients and was small in 77.8 % of the patients. The mean pleural fluid WBC count was 1619.7 ± 1432/mm^3^, and the differential count showed a predominance of mononuclear cells in 11 patients. The mean pleural fluid protein and LDH levels were 2.30 ± 0.58 g/dL (range, 1.26–3.16 g/dL) and 275.8 ± 101.6 U/L (range, 82–416 U/L), respectively and were slightly more elevated than normal pleural fluid. The mean pleural fluid pH was 7.43 ± 0.82 (range, 7.16–7.58). The mean pleural fluid ADA level was 32.5 ± 20.9 U/L; it was greater than 35 U/L in 8 patients (44.4 %) and over 70 U/L in 2 of these patients.

**Table 4:** Characteristics and laboratory findings of pleural effusion in scrub typhus (18 patients)

Characteristics	PE underwent thoracentesis (*n* = 18)
Time from disease onset to thoracentesis, mean days ± SD	13.17 ± 13.11
Time from admission to thoracentesis, mean days ± SD	2.50 ± 2.57
Location (%)	
Unilateral right sided	0 (0.0)
Unilateral left sided	0 (0.0)
Bilateral	18 (100.0)
Size (%)	
Small	14 (77.8)
Moderate	4 (22.2)
Large	0 (0.0)
Laboratory findings, mean ± SD	
WBC count (no. of cells/mm^3^)	1619.7 ± 1432.4
Polymorphonuclear leukocytes (%)	45.1 ± 28.4 (*n* = 9)
Mononuclear cell mean percentages (%)	54.9 ± 28.4 (*n* = 11)
Total protein	
Serum (g/dL)	5.83 ± 0.70
Pleural fluid (g/dL)	2.30 ± 0.58
Pleural fluid/serum ratio	0.39 ± 0.80
LDH	
Serum (U/L)	614.7 ± 228.0
Pleural fluid (U/L)	275.8 ± 101.6
Pleural fluid/serum ratio	0.46 ± 0.12
Pleural fluid/upper limit of normal for serum LDH ratio	0.81 ± 0.37
Albumin	
Serum (g/dL)	3.03 ± 0.44
Pleural fluid (g/dL)	1.45 ± 0.40 (*n* = 15)
Serum-Pleural fluid albumin gradient	1.58 ± 0.40 (*n* = 15)
pH	7.43 ± 0.82
Glucose (mg/dL)	139.4 ± 75.9
ADA (U/L), mean ± SD	32.5 ± 20.9
< 35, no. of patients (%)	10 (55.6)
35–70, no. of patients (%)	6 (33.3)
> 70, no. of patients (%)	2 (11.1)
CEA (ng/ml)	3.64 ± 3.15

*Abbreviations*: *LDH* lactate dehydrogenase, *ADA* adenosine deaminase, *CEA* carcinoembryonic antigen

### Separation of exudate from transudate of pleural effusion in scrub typhus patients

The pleural effusion had the characteristics of transudate in 7 patients and exudate in 11 patients by Light’s criteria. All of the exudates satisfied the criteria of pleural fluid with an LDH level greater than two thirds of the upper limit of normal serum LDH. The criteria of a PF-to-S protein ratio > 0.5 and a PF-to-S LDH ratio > 0.6 were each satisfied by two patients (11.1 %). The features of the examined pleural fluid are shown in Table 5.

**Table 5:** Characteristics of pleural effusion in scrub typhus

Pt	Comorbidities	Other Cx.	Radiographic findings	Size/Side	pH	RBC count	WBC count	PMNL (%)	MNC (%)	PF Pro	Pro ratio	PF LDH	LDH ratio	UNL ratio	PF Alb	SEAG	Glu	ADA	
1	-	-	IP, Edema, PE, Cardiomegaly	S/B	7.389	-	1900	90	10	2.08	0.38	267	0.32	0.58	-	-	81	22	T
2	-	AKI	IP, Edema, PE	S/B	7.415	-	2640	55	45	2.81	0.42	287	0.45	0.62	-	-	148	78	T
3	-	Pneumonia	IP, Edema, PE, Consolidation	S/B	7.360	-	2070	40	60	2.68	0.43	393	0.35	0.85	1.44	1.46	90	49	E
4	-	AKI, A fib., UGI bleeding	IP, Edema, PE, Cardiomegaly	S/B	7.455	-	713	30	70	2.53	0.47	282	0.52	0.61	1.69	1.21	130	36	T
5	HTE,	Pneumonia	IP, Edema, PE, Consolidation	S/B	7.388	75	864	25	75	2.35	0.38	281	0.44	0.61	1.33	1.40	206	40	T
6	DM	Cholecystitis	IP, Edema, PE	S/B	7.451	108	658	25	75	1.71	0.33	416	0.46	0.90	1.22	1.64	118	47	E
7	-	A fib., SCMP	IP, Edema, PE	M/B	7.581	-	120	40	60	1.94	0.38	140	0.32	0.30	1.29	1.55	135	23	T
8	DM, DCMP	-	IP, Edema, PE, Cardiomegaly	M/B	7.449	-	147	30	70	2.61	0.45	325	0.45	0.71	1.98	1.77	272	21	E
9	-	-	IP, Edema, PE	S/B	7.433	-	2008	10	90	2.10	0.36	170	0.33	0.81	1.37	1.42	99	16	E
10	HTE	Pneumonia	PE, Consolidation	S/B	7.472	-	1231	15	85	2.51	0.37	123	0.59	0.58	-	-	111	2.3	T
11	-	Pneumonia	IP, Edema, PE, Consolidation	M/B	7.162	-	3980	30	70	2.96	0.53	360	0.63	1.71	1.95	1.15	17	78.7	E
12	-	-	IP, PE	S/B	7.417	-	4492	90	10	1.52	0.28	170	0.36	0.81	1.04	1.90	92	22.5	E
13	HTE, DM	AKI	IP, Edema, PE	M/B	7.460	10,000	72	25	75	1.47	0.31	244	0.46	1.16	0.93	1.49	132	16.2	E
14	-	-	IP, Edema, PE	S/B	7,478	-	1166	10	90	3.17	0.42	343	0.73	1.63	2.16	1.88	70	36.8	E
15	DM, LC, HCC	-	IP, PE	S/B	7.455	390,000	550	55	45	1.26	0.23	82	0.29	0.39	0.88	2.56	315	9.4	T
16	Breast Ca.	-	IP, Edema, PE	S/B	7.439	-	4492	90	10	2.63	0.49	322	0.53	0.70	1.87	1.31	99	20.0	E
17	HTE, DM, LC	AKI	IP, Edema, PE	S/B	7.428	-	1037	85	15	1.82	0.32	406	0.43	0.88	1.13	1.98	143	26.2	E
18	HTE, DM, LC	Meningitis	IP, Edema, PE	S/B	7.480	-	1015	70	30	3.16	0.51	353	0.56	0.77	1.53	0.93	251	41.2	E

*Abbreviations*: *Pt* patient, *Gen* gender, *THE* hypertension, *DM* diabetes mellitus, *DCMP* dilated cardiomyopathy, *LC* liver cirrhosis, *HCC* hepatocellular carcinoma, *Cx.* complications, *AKI* acute kidney injury, *A fib.* atrial fibrillation, *UGI* upper gastrointestinal, *IP* interstitial pneumonitis, *PE* pleural effusion, *PF* pleural fluid, *S* small, *M* moderate, *B* bilateral, *PMNL* polymorphonuclear leukocytes (%), *MNC* Mononuclear cell (%), *Pro* protein, *ratio* PF/serum ratio, *LDH* lactate dehydrogenase, *UNL ratio* PF/upper limit of normal for serum LDH ratio, *Alb* albumin, *SEAG* serum-pleural fluid albumin gradient, *Glu* glucose, *ADA* adenosine deaminase, *T* transudate, *E* exudate

## Discussion

In our retrospective study, the chest radiographs were routinely examined when the patients were initially admitted, and 51.2 % of the chest radiographs of the confirmed adult scrub typhus patients had abnormal findings. The abnormal findings included the following: interstitial pneumonia (39.8 %), pulmonary alveolar edema (16.9 %), pleural effusion (20.9 %), and cardiomegaly (15.1 %); these findings were not significantly different from previously reported results [5–9]. Compared with previous studies, this report provides the first information on the factors associated with the occurrence of pleural effusion and the characteristics of pleural effusion in adult scrub typhus.

The principal pathophysiological findings of scrub typhus are widespread vasculitis or perivasculitis [12]. Multiplication of the organism in microvascular endothelial cells causes endothelial cell proliferation and perivascular inflammatory cell infiltration in multiple organs, including the lungs [12]. These microangiopathies alter capillary permeability and lead to noncardiogenic pulmonary interstitial edema, which is a component of interstitial pneumonia [4, 6]. Additionally, alteration of the relationship of the hydrostatic and oncotic forces favoring fluid filtration into the lung secondary to cardiac dysfunction and hypoalbuminemia could be associated with the cause of interstitial pneumonia, a common radiographic finding of scrub typhus [5, 6]. As such, the basic pathological process of pulmonary involvement in scrub typhus is interstitial pneumonia with or without vasculitis. Similarly, pleural effusion in scrub typhus most likely developed because of an exudative and/or transudative process. In a Rocky Mountain spotted fever report, pleural effusion had transudate or exudate characteristics [13]. Additionally, Song et al showed that interstitial pneumonia was accompanied with pleural effusion, cardiomegaly and pulmonary alveolar edema in the majority of cases [6]. These reports support the assumption of the pathogenesis of pleural effusion in scrub typhus.

In this study, older age, male gender, the presence of heart failure and lower albumin were found by multivariate analysis to be independently predictive variables for the occurrence of pleural effusion in scrub typhus.

According to another retrospective study, elderly patients had more complications such as acute kidney injury, pneumonia and septic shock than younger patients, and a linear trend was observed between age and complication rates [14]. Additionally, typical chronic illnesses in elderly patients such as cardiovascular disease contribute to complications [14]. Kim et al reported that age over 60 was independently associated with severe scrub typhus [15]. In our study, 77 patients (82.8 %) in the group with effusion were >65 years of age, and most of them had at least one underlying disease. It is possible that older scrub typhus patients had a greater incidence of comorbid illnesses, increased complications and a more severe disease course, which led to a greater occurrence of pleural effusion. This relationship remains unclear.

Hypoalbuminemia, defined as a first serum albumin of less than 3.0 g/dL on the initial patient visit, could be observed in one-fourth to three-fourths of scrub typhus patients [6, 16]. Although the pathogenetic mechanism of hypoalbuminemia in adult scrub typhus patients is not completely known, it is considered to be associated with the widespread transvascular leakage of protein resulting from altered permeability [17]. Min et al first demonstrated the loss of protein via the intestinal tract in scrub typhus patients by abdominal scintigraphy using technetium 99-m labeled human serum albumin and fecal clearance of alpha1- antitrypsin [18]. The presumed mechanism for intestinal albumin loss is the increase in permeability resulting from vasculitis of the small vessels. Additionally, there has been reported that in patients with chronic diseases (e.g., congestive heart failure, liver cirrhosis and malignancy), in whom hypoalbuminemia resulting from decreased hepatic production of albumin is frequently the cause of pleural effusion. When the pleural microvasculature is damaged in exudative processes, the level of albumin leakage into the pleural cavity progressively increases depending on the degree of injury. The serum-effusion albumin gradient (SEAG) is highest in transudative effusions because less albumin is filtered through the relatively normal pleural microvasculature. In our study, the mean serum albumin level of the group with effusion was 3.11 ± 0.51 g/dL, which was lower than that of the group without effusions, and 45 patients (48.4 %) had hypoalbuminemia (≤3.0 g/dL). The mean SEAG observed in 15 patients was 1.58 ± 0.40 g/dL (range, 0.93–2.56 g/dL), and SEAG < 1.2 g/dL, indicating an exudative process, occurred in two patients. One plausible explanation for this result is that the degree of microvascular injury in the lungs and pleura might not be severe or that the transudative process resulting from decreased oncotic pressure might contribute to the formation of effusion. Hypoalbuminemia with or without increased vascular permeability might cause the effusions.

In congestive heart failure, the capillary hydrostatic pressure might be increased because of the elevated left-sided filling pressure observed with a fluid overload. In our study, the patients with effusion had a significantly higher occurrence of heart failure. Additionally, the transudative process resulting from increased hydrostatic pressure as well as hypoalbuminemia might contribute to the formation of effusion.

In this study, we performed thoracentesis in 18 patients, and the demographics, clinical characteristics, chest radiographic abnormalities and pleural fluid data of the patients are shown in Table 5. The fluid from the patients who underwent thoracentesis had transudate and exudate characteristics based on Light’s criteria. Eleven effusions revealed mononuclear cell predominance in the differential count. In our laboratories, we divided pleural fluid WBCs into polymorphonuclear leukocytes and mononuclear cells; however, we did not provide additional information regarding the cell types (e.g., lymphocytes, eosinophils). Relatively lower mean levels of LDH and protein than those of other infectious diseases such as bacterial pneumonia, tuberculosis and Rocky Mountain spotted fever reported [13]. Additionally, 2 patients had a pleural fluid protein level >3.0 g/L, which suggests exudate. The mean pleural fluid pH was slightly lower than the range of normal pleural fluid. Except in one patient (No.11), the pleural fluid pH was above 7.35, and the pleural fluid glucose was greater than 60 mg/dL. None of the effusions had positive Gram staining or culture findings. In the case of patient No. 11, the effusion was complicated parapneumonic effusion based on Light’s classification because the pleural fluid pH was 7.16 and the pleural fluid glucose was 17 mg/dL [19]. The chest radiograph findings for patient No. 11 showed obvious pneumonic infiltration of both lower lung fields. The mean pleural fluid ADA was over 35 U/L in 8 patients (44.4 %), and in 2 of these patients, it was over 70 U/L. ADA levels are particularly useful in areas where the prevalence of tuberculosis is high. An ADA level greater than 35 U/L has a sensitivity of approximately 93 % and a specificity of approximately 90 % for the presence of tuberculosis [20]. A future comparative study with tuberculous effusion is required.

There are some limitations in our study. First, this study was a retrospective survey, which resulted in incomplete data, and it did not control the laboratory and radiologic examinations of all the adult scrub typhus patients. Second, only 18 cases of scrub typhus were studied. The fluid from some patients who had more than a 10-mm thickness of pleural fluid and the fluid from the patients who had insignificant effusions were not analyzed, which might have limited the interpretative power of the results.

The study provides the first information regarding the factors associated with the occurrence of pleural effusion and the characteristics of pleural effusion in adult scrub typhus. Follow-up investigations or prospective evaluations should be conducted for further information.

## Conclusions

This study determined the factors associated with the occurrence of pleural effusion and the characteristics of pleural effusion in adult scrub typhus. Clinicians should suspect for occurring of pleural effusion when scrub typhus patients are older and male, with underlying heart failure and hypoalbuminemia. The presentations of pleural effusion in scrub typhus patients were bilateral and small in most cases, with transudate and/or exudate characteristics.

## Abbreviations

ADA, adenosine deaminase; ALP, alkaline phosphatase; ALT, alanine aminotransferase; AST, aspartate aminotransferase; CEA, carcinoembryonic antigen; CI, confidence interval; CRP, C-reactive protein; dL, deci-litre; ESR, erythrocyte sedimentation rate; g, gram; IFA, immunofluroescent antibody; IgM, immunoglubolin M; IU, international unit; LDH, lactate dehydrogenase; PF: pleural fluid; SCr, serum creatinine; WBC: white blood cell;
